# Chromosomal Abnormalities and Pregnancy Outcomes for Fetuses With Gastrointestinal Tract Obstructions

**DOI:** 10.3389/fped.2022.918130

**Published:** 2022-06-06

**Authors:** Xiaoqing Wu, Linjuan Su, Qingmei Shen, Qun Guo, Ying Li, Shiyi Xu, Na Lin, Hailong Huang, Liangpu Xu

**Affiliations:** ^1^Department of Medical Genetic Diagnosis and Therapy Center, Fujian Maternity and Child Health Hospital, College of Clinical Medicine for Obstetrics and Gynecology and Pediatrics, Fujian Medical University, Fuzhou, China; ^2^Fujian Provincial Key Laboratory for Prenatal Diagnosis and Birth Defect, Fuzhou, China; ^3^Department of Laboratory Medicine, Fujian Medical University, Fuzhou, China; ^4^Department of Pediatrics, Guangxi Medical University, Nanning, China

**Keywords:** gastrointestinal tract obstructions, traditional karyotyping, chromosomal abnormalities, chromosomal microarray analysis, Down syndrome, copy number variants

## Abstract

Fetal gastrointestinal tract obstruction (GITO) is the most frequently encountered gastrointestinal defect in the prenatal period. This study aimed to investigate the genetic disorders and pregnancy outcomes of fetal GITO. We reviewed data from 70 pregnancies that were referred for invasive prenatal testing because of fetal GITO. According to the level of obstruction, they were classified into esophageal atresia/stenosis, duodenal atresia/stenosis, jejunal or ileal atresia/stenosis, or anal atresia. Traditional karyotyping was performed on all the 70 pregnancies, and chromosomal microarray analysis (CMA) was performed on 32 of them in parallel. Traditional karyotyping revealed twelve (17.1%) chromosomal abnormalities, including 11 cases of trisomy 21 (Down syndrome), and one case of a supernumerary marker chromosome related to Cat eye syndrome. According to the absence or presence of other ultrasound anomalies, they were categorized into isolated GITO (*n* = 36) and non-isolated GITO (*n* = 34). The rate of chromosomal abnormalities in the non-isolated GITO pregnancies was significantly higher than that in the isolated GITO pregnancies (29.4 vs. 5.5%, *p* < 0.05); the survival rate in the isolated group was significantly higher than that in the non-isolated group (67.6 vs. 34.4%, *p* < 0.05). Among the 32 cases where CMA was performed, an additional one (3.1%) copy number variant with clinical significance was noted in a fetus with normal karyotype. The microduplication on 7q12 was considered to be the genetic etiology of duodenal stenosis, although it was inherited from a phenotypically normal mother. Our study supports the strong association between Down syndrome and fetal GITO, especially duodenal stenosis. Our findings suggested that the risk of chromosomal abnormalities was increased when GITO was accompanied by other ultrasound anomalies; thus, chromosomal abnormalities and fetal anatomy should be carefully evaluated for pregnancy management of fetal GITO.

## Introduction

Fetal gastrointestinal tract obstruction (GITO) is the most frequently encountered gastrointestinal defect in the prenatal period. It is the result of maldevelopment of the gastrointestinal tract including atresia and stenosis and is reported to affect one–five per 100,00 live births (1–3). It is mainly diagnosed by routine obstetrical ultrasound screening during the second and third trimesters with variable diagnostic accuracy (1, 4, 5). Indirect ultrasound findings such as failure to visualize the stomach, double bubble, and dilated bowel loops are typical signs. The obstruction may occur at any site of the gastrointestinal tract. According to different levels of obstruction, they are generally defined as esophageal atresia/stenosis, duodenal atresia/stenosis, jejunal or ileal atresia/stenosis, or anorectal malformation.

The etiology and pathogenesis of GITO are not completely understood, because genetic, biological, hormonal, and environmental factors all play an important role in it (5–8). Medical care such as surgical correction within the first few days after birth can effectively improve the prognosis; thus, most infants with congenital GITO tend to have an excellent prognosis (9, 10). However, the premise is that there are no other ultrasound anomalies and chromosomal abnormalities. GITO could be isolated or more frequently accompanied by other ultrasound anomalies, which were reported to present in 10%−65% of cases (1, 2, 11, 12), especially in esophageal and anorectal obstruction cases. The association between GITO and chromosomal abnormalities has been explored in several previous reports (13). For instance, trisomy 21, trisomy 18, and trisomy 13 have been observed in cases with esophageal atresia (14, 15). Best et al. (16) reported that 20% of small intestinal atresia cases were associated with chromosomal anomalies. However, most of these data were from traditional karyotyping, whereas the detective efficiency of chromosomal microarray analysis (CMA) in GITO is limited. Here, we presented our experience on prenatal diagnosis of GITO based on genetic disorders by traditional karyotyping and SNP array testing, as well as associated ultrasound abnormalities, to provide more evidence for genetic counseling and pregnancy management.

## Materials and Methods

### Patients and Samples

Between May 2012 and November 2021, a total of 73 singleton pregnancies were referred for invasive prenatal testing because of fetal digestive tract atresia/stenosis at the Medical Genetic Diagnosis and Therapy center of Fujian Maternal and Child Health Hospital, China. Three cases were excluded because GITO was not observed on repeat prenatal ultrasound. As a result, 70 pregnancies were enrolled, including nine cases of esophageal atresia/stenosis, 43 cases of duodenal atresia/stenosis, 16 cases of jejunal or ileal atresia/stenosis, and two cases of anal atresia. All the diagnoses of GITO were made by ultrasonography, and 15 of them were confirmed further by magnetic resonance imaging (MRI). The gestational age at the diagnosis of GITO was 26.3 ± 3.6 weeks. The general information is presented in Table 1.

**Table 1:** Demographic characters for 70 pregnancies with fetal gastrointestinal tract obstruction (GITO).

	**Isolated GITO (*n* = 36)**	**Non-isolated GITO (*n* = 34)**	**Total**
Maternal age (years), (range, median, mean ± SD)	23–40, 28, 29.0 ± 4.7	20–39, 30, 30.2 ± 5.0	20–40, 29, 29.6 ± 4.8
Gestation age at GITO initially diagnosed (Range, Median, Mean ± SD)	21–32, 26.5, 26.3 ± 3.6	15–33, 25, 25.5 ± 4.0	15–33, 25.5, 25.9 ± 3.7
>16, ≤ 28 weeks (*n*, %)	25, 69.4%	27, 79.4%	52, 74.3%
>28 weeks (*n*, %)	11, 30.6%	7, 20.6%	18, 25.7%
Specimen
AF (*n*, %)	16, 44.4.0%	19, 55.9%	36, 51.4%
CB (*n*, %)	20, 55.6%	15, 44.1%	34, 48.6%
Type of GITO
Esophageal atresia/stenosis (*n*, %)	3, 10.0%	6, 17.6%	9, 12.9%
Duodenal atresia/stenosis (*n*, %)	24, 66.7%	19, 55.9%	43, 61.4%
Jejunal or ileal atresia/stenosis (*n*, %)	9, 25.0%	7, 20.6%	16, 22.9%
Anal atresia (*n*, %)	0, 5.0%	2, 5.9%	2, 2.9%

*AF, amniotic fluid; CB, cord blood*.

The enrolled cases were categorized into non-isolated GITO (*n* = 36) and isolated GITO (*n* = 34) according to the presence or absence of other ultrasound abnormalities. Considering that polyhydramnios was a frequent development secondary to gastrointestinal obstruction, GITO accompanied with polyhydramnios was categorized as isolated GITO.

Generally, taking into account the risk of invasive procedures and the success rate of cell culture, we offered an amniocentesis to cases with gestational ages within 24 weeks, and cordocentesis to cases with gestational age beyond 24 weeks. As a result, the prenatal specimens included 36 cases of amniotic fluid and 34 cases of cord blood. Traditional karyotyping was performed on all 70 pregnancies, and an SNP array was performed in parallel on 32 of them.

Follow-up assessments were performed *via* clinical records or telephone calls, and the age at follow-up ranged from 3 months to 10 years. The study was approved by the local Ethics Committee of Fujian Maternity and Child Health Hospital. Written informed consent to participate in the study was obtained from each patient.

### Traditional Karyotyping

Traditional karyotyping consisted of cell culture, and G-banded karyotyping was performed on cultured amniotic fluid or fetal cord blood according to the standard protocols in our laboratory. The karyotype was determined at a resolution of 320–500 band level.

### CMA Platforms and Data Interpretation

Genomic DNA was extracted from uncultured amniotic fluid or fetal cord blood using a QIAGEN kit (Qiagen, Hilden, Germany) and according to the manufacturer's instructions. A single nucleotide polymorphism (SNP) array was performed using an Affymetrix CytoScan 750K array (Affymetrix Inc., Santa Clara, CA, United States), which included 200,000 probes for SNPs and 550,000 probes for copy number variants (CNVs) distributed across the entire human genome. The Chromosome Analysis Suite software (Affymetrix) and human genome version GRCh37 (hg19) were used. A resolution was generally applied: gains or losses of ≥400 kb and loss of heterozygosity (LOH) ≥10 Mb. All detected CNVs were compared with in-house and national public CNV databases as follows: Database of Genomic Variants (DGV), Database of Chromosome Imbalance and Phenotype in Humans Using Ensemble Resources (DECIPHER), International Standards for Cytogenomic Arrays Consortium, and Online Mendelian Inheritance in Man (OMIM). The CNVs were classified into five groups according to the American College of Medical Genetics (ACMG) definitions (17) and local database: pathogenic, benign, likely pathogenic, likely benign, and variant of unknown significance (VOUS). Pathogenic/likely pathogenic CNVs were considered clinically significant findings. CNV inherited from a phenotypically normal parent was considered to be likely benign.

### Statistical Analysis

The data were analyzed with SPSS software v26.0 (SPSS Inc., Chicago, IL, United States). Statistical comparisons were performed by chi-square test, and *p* < 0.05 was considered statistically significant.

## Results

### Results of Traditional Karyotyping

The traditional karyotyping revealed 12 (17.1%) chromosomal abnormalities from a total 70 cases (Table 2). The detection rates in pregnancies of esophageal atresia/stenosis, duodenal atresia/stenosis, jejunal or ileal atresia/stenosis, and anorectal malformation were 11.1, 23.3, 0, and 50%, respectively (Table 3). The aberrations included 11 cases of trisomy 21 (T21) and 1 case of supernumerary marker chromosome (sSMC). Among them, T21 was noted in 10 out of the 43 (23.3%) cases of duodenal atresia/stenosis (cases 1–2 and 4–11, Table 2) and in one out of the nine (11.1%) cases of esophageal atresia/stenosis (case 12). For sSMC analysis (case 13), the SNP array demonstrated four copies of the gene dosage encompassing the 22q11.1q11.21 region (16,888,899–18,649,190). FISH studies were subsequently performed using a chromosome 22-specific DNA probe and showed that the marker had two 22q11.2 signals. Therefore, the karyotype was finally confirmed to be 47, XY,+psuidic(22) (q11.2), harboring additional copies of cat eye syndrome (CES, OMIM # 115470) critical region genes. The affected fetus showed suspected anal atresia and multiple malformations. The parents declined to terminate the pregnancy, and the fetus was delivered at 38 gestational weeks but died 10 days after birth.

**Table 2:** Details of 12 abnormal karyotypes and one copy number variants.

**Case number**	**Maternal age (years)**	**Gestational age at ultrasound diagnosis**	**Specimens**	**Ultrasound findings**	**Karyotype**	**CMA results**	**Outcome**
**Isolated group**							
1	40	22+	AF	Duodenal atresia/stenosis	47,XY,+21	arr[GRCh37](21) ×3	TOP
2	35	27+	CB	Duodenal atresia/stenosis	47,XX,+21	/	TOP
3	31	22	AF	Duodenal stenosis	46,XY	arr[GRCh37] 17q12 (34,440,088–36,243,365) ×3 mat	TOP
**Non-isolated group**							
4	33	21+	AF	Duodenal atresia/stenosis, VSD, agenesis of corpus callosum, bilateral ventriculomegaly, increased NT, nasal bone dysplasia, aberrant right subclavian artery	47,XY,+21	/	TOP
5	35	22+	AF	Duodenal atresia/stenosis, VSD	47,XX,+21	/	TOP
6	30	22+	AF	Duodenal atresia/stenosis, VSD, pulmonary stenosis, nasal bone dysplasia	47,XY,+21	arr[GRCh37](21) ×3	TOP
7	32	28+	CB	Duodenal atresia/stenosis, polyhydramnios, bilateral hyperechoic kidneys	47,XY,+21	/	TOP
8	38	32+	CB	Duodenal atresia/stenosis, polyhydramnios, short femur, short humerus	47,XY,+21	/	TOP
9	28	18+	AF	Duodenal atresia/stenosis, increased NT	47,XY,+21	/	TOP
10	28	24+	AF	Duodenal atresia/stenosis, cardiac malformation	46,XX,rob(14,21)(q10;q10),+21	/	TOP
11	39	24+	AF	Esophageal atresia/stenosis, EIF, short femur, short humerus	46,XY,+21	/	TOP
12	33	30+	CB	Duodenal atresia/stenosis, VSD, aortic stenosis, tricuspid regurgitation	47,XX,+21	/	TOP
13	37	21+	AF	Anal atresia, polyhydramnios, VSD, double superior vena cava, aberrant right subclavian artery, right ventriculomegaly, single umbilical artery	47,XY,+psu idic(22)(q11.2)	arr[GRCh37] 22q11.1q11.21(16,888, 899–18,649,190) ×4	Died 10 days after birth

*AF, amniotic fluid; CB, cord blood; VSD, ventricular septal defect; NT, nuchal thickness; EIF, echogenic intracardiac focus; TOP, termination of pregnancy*.

**Table 3:** Distribution of chromosomal abnormalities in different types of GITO.

	**Traditional**	**SNP array analysis (*****n*** **=** **32)**	
	**karyotyping** **(*n* = 70)**	**Karyotype detectable**	**Karyotype undetectable**
Esophageal atresia/stenosis	1, 11.1%	0, 0.0%	0, 0.0%
Duodenal atresia/stenosis	10, 23.3%	2, 11.8%	1, 5.9%
Jejunal or ileal atresia/stenosis	0, 0.0%	0, 0.0%	0, 0.0%
Anal atresia	1, 50.0%	1, 50%	0, 0.0%
Total	12, 17.1%	3, 9.4	1, 3.1%

The rate of chromosomal abnormalities determined by traditional karyotyping in isolated GITO was 5.5% (2/34), which is significantly lower than the 29.4% (10/24) in the non-isolated group (*p* < 0.05). Polyhydramnios was the most common accompanying abnormality that was noted in 41.4% (29/70) of the cases, followed by cardiovascular malformations (11.4%, 8/70) including atrioventricular septal defect, aortic stenosis, pulmonic stenosis, and persistent left superior vena cava.

### CMA Results of 32 Fetuses

**Figure 1:**
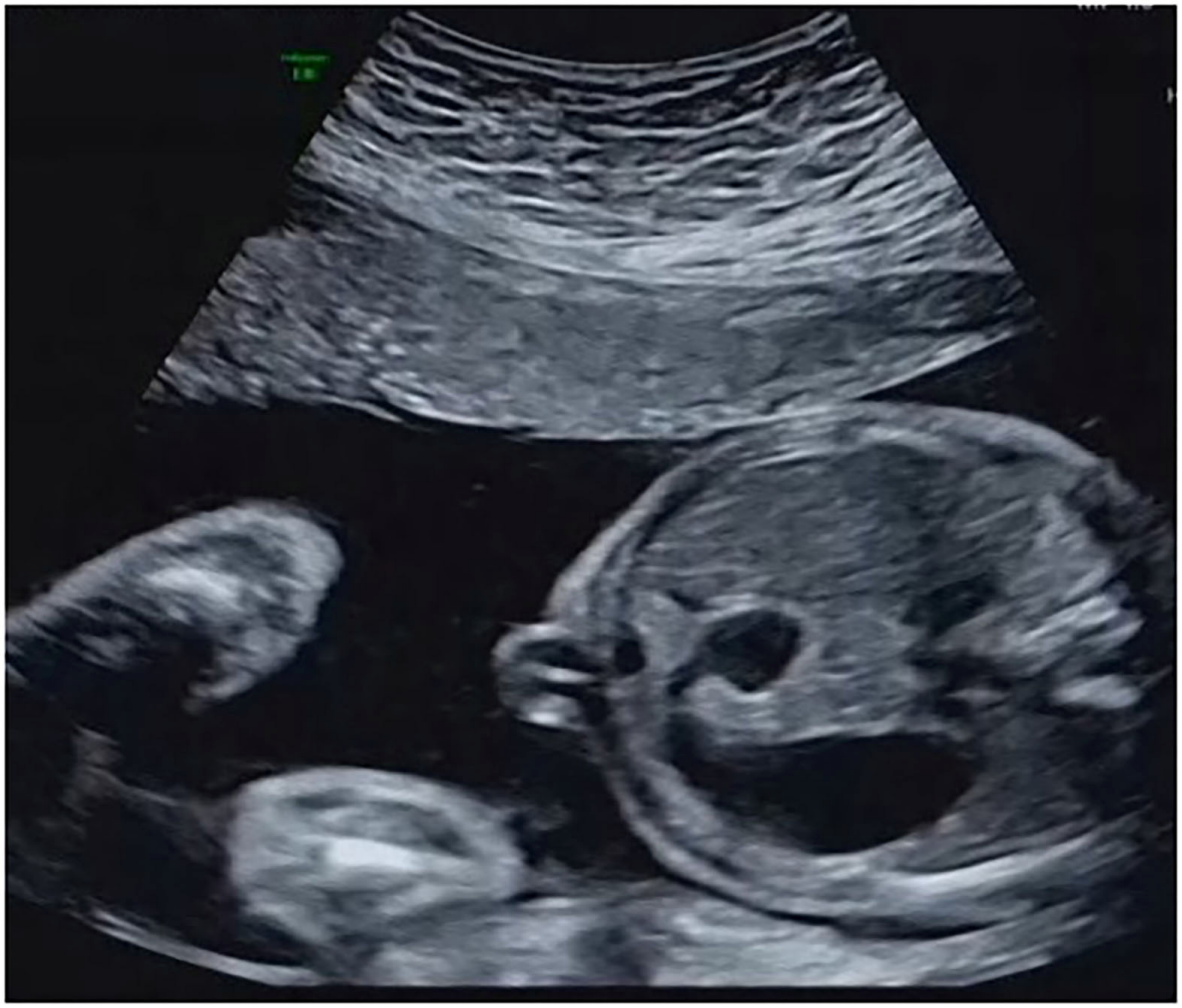
The presence of the “double-bubble” sign.

**Figure 2:**
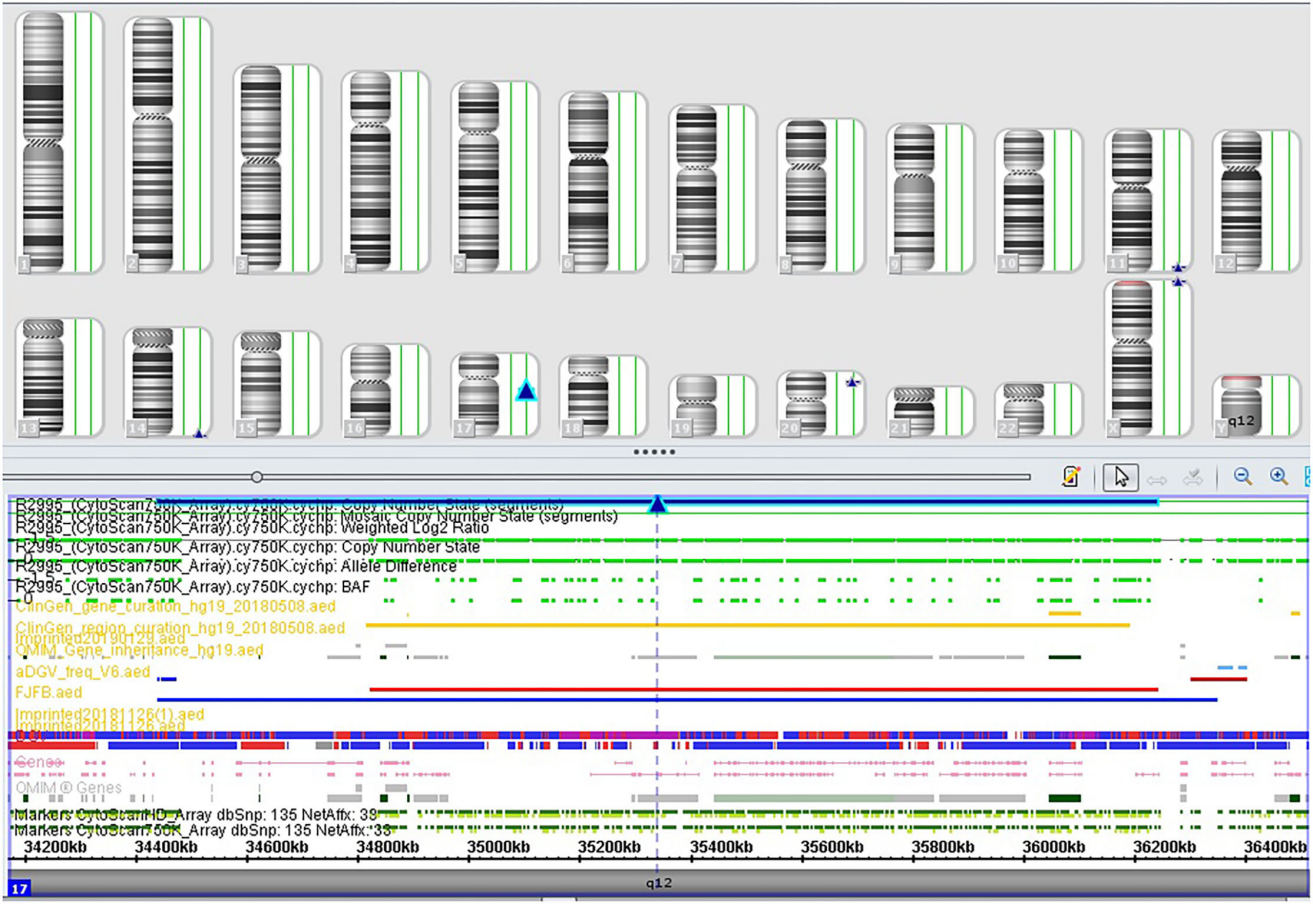
SNP array profile of Case 3. The image indicates a 1.8 Mb duplication on 17q12 (34,440,088_36,243,365).

Among the 32 cases that underwent traditional karyotyping and CMA in parallel, an additional pathogenic aberration (case 3, Table 2) was identified in a fetus with duodenal atresia/stenosis (Figure 1) and normal karyotype, contributing to an incremental detection yield of 5.9% (1/17) for fetuses with duodenal atresia. The fetus had a 1.8-Mb duplication in the 17q12 region (Figure 2), which is responsible for17q12 duplication syndrome (OMIM # 614526). The overall penetrance of the syndrome for developing any disorder was reported to be nearly 21.1%. Here, the CNVs were categorized as “pathogenic,” although it was inherited from a phenotypically normal mother. The pregnancy was terminated. The detailed ultrasound findings, CMA results, and pregnancy outcomes are summarized in Supplementary Table 1.

### Clinical Follow-Up

Follow-up information was obtained for 66 (94.3%) of the cases. The overall survival rate and TOP rates were 51.3 and 33.3%, respectively. Twenty-two of the cases ended in pregnancy termination, and three were stillbirth. Among the 41 retained pregnancies, three cases had normal MRI and no gastrointestinal obstruction syndrome after birth; 37 neonates underwent surgery operation, but three of them died within 6 months because of other complications, and 34 of them had normal development during the follow-up period; the remaining case (case 13) died 10 days after birth owing to anal atresia and cardiac malformation. The details are presented in Table 4. The survival rate in the isolated group was significantly higher than that in the non-isolated group (67.6 vs. 34.4%, *p* < 0.05), and duodenal atresia/stenosis had the highest survival rate (61.9%) among the other levels of GITO.

**Table 4:** Follow-up information for 66 pregnancies with GITO.

	**Total**	**Isolated GITO**	**Non-isolated GITO**	**Esophageal atresia/stenosis**	**Duodenal atresia/stenosis**	**Jejunal or ileal atresia/stenosis**	**Anal atresia**
TOP	22, 33.3%	7, 20.6%	15, 46.9%	3, 33.3%	14, 33.3%	5, 38.5%	0, 0.0%
Stillbirth	3, 4.5%	1, 2.9%	2, 6.3%	1, 11.1%	1, 2.4%	1, 7.7%	0, 0.0%
Normal development after surgery	34, 51.5%	23, 67.6%	11, 34.4%	4, 44.4%	26, 61.9%	3, 23.1%	1, 50.0%
Infant death	4^a^, 6.1%	1, 2.9%	3, 9.4%	0, 0.0%	1, 2.4%	2, 15.4%	1, 50.0%
Misdiagnosis	3^b^, 4.5%	2, 5.9%	1, 3.1%	1, 11.1%	0, 0.0%	2, 15.4%	0, 0.0%
Total	66, 100.0%	36, 100%	34, 100%	9, 100%	42, 100.0%	13, 100%	2, 100.0%

*^a^Three cases died several months after surgery; one case died 10 days after birth*.

*^b^Three cases revealed normal MRI and no gastrointestinal obstruction syndrome after birth*.

## Discussion

Consistent with most studies (18), almost all cases of fetal digestive tract atresia/stenosis were diagnosed in the second and third trimesters. Owing to late gestational age at diagnosis, GITO is a difficult condition for clinicians to treat. In general, once fetal GITO is suspected, chromosomal analysis and thorough ultrasound examination are strongly recommended for fetal prognosis assessment and pregnancy management.

Most previous reports focused on cytogenetic aberrations, with frequencies ranging from 5.4 to 20% (1, 2, 5, 15, 16, 19, 20). The association between GITO and aneuploidies, such as T21 and T18, has been well-established. In our study, the traditional karyotyping identified chromosomal abnormalities in 21.4% of the cases, including11 cases of T21 and a single case of 22q11.2 partial tetrasomy. Among them, 10 cases of T21 were observed in fetuses with duodenal stenosis, accounting for 23.3% of all the pregnancies with duodenal stenosis, which is considerably lower than the proportion reported by Tonni et al. (5) and Bethell et al. (3). This is probably attributed to improvements in the quality of ultrasound evaluation, indicating T21, and non-invasive prenatal testing (NIPT) that screen T21 with high specificity and sensitivity and detect it earlier. Consequently, more cases of pregnancy with identified T21 have been terminated before GITO could be detected by ultrasound scans. Esophageal atresia/stenosis is more frequently reported to be associated with T18 (2, 11), and this association may be explained by the decreased cholesterol synthesis observed in a series of neonates and fetuses with T18 (21, 22). Owing to the small size of the sample, our data could not confirm this relationship, but only one case of T21 was noted in a fetus with esophageal atresia/stenosis that was accompanied by soft markers. Among the different levels of GITO, jejunum and ileum atresia/stenosis is known to have the lowest risk of chromosomal anomalies (1, 23). In this study, no abnormalities were detected in the jejunum or ileum atresia/stenosis. Overall, our results further support the close association of DS and GITO, especially in cases of duodenal and esophageal stenoses. Efforts have been made to identify the contributing factors of congenital defects among Down Syndrome fetuses (24, 25), but the exact mechanism has yet to be explained. In addition to T21, a rare aberration was noted in a fetus with anal atresia, which has been described in our previous case report (26). The CES derived from duplicated regions of 22pter-22q11.2 is characterized clinically by the combination of coloboma of the iris and multiple malformations. Anal atresia is one of the most common gastrointestinal malformations, occurring in approximately 73%−81% of CES cases (27), but it is rarely detected in fetuses with anal atresia probably because of the difficulty in identifying prenatal anal atresia. Confirmation of CES requires the assistance of molecular methodologies such as FISH or CMA.

With the wide application of CMA in prenatal diagnosis, some pathogenic CNVs have been identified in fetal GITO (28, 29). It is well-known that CMA produces a significant yield of clinically relevant abnormalities in pregnancies with various ultrasonographic anomalies and normal karyotype (30, 31). In this study, we observed an additional 5.9% of pathogenic aberration by SNP array in pregnancies with duodenal atresia/stenosis, which is lower than that reported by Bishop et al. (32) and Zhang et al. (33). The divergence might be attributed to different sample sizes. The only CNV in our study showed a 1.8-Mb microduplication at 17q12 that overlapped with the critical region of 17q12 duplication syndrome (OMIM # 614526). The HNF1B gene in 17q12 is a transcription factor that is expressed during endoderm and mesoderm development, and it plays an important role in organ differentiation of the genitourinary and gastrointestinal tracts. Mutation in the HNF1B gene might be responsible for the range of phenotypes because of its essential role in early human GI development (34, 35). The syndrome has been regarded as a pathogenic variant with an incomplete penetrance of 22.1% (36, 37), which explained why the mother harbored the same aberration but had a normal phenotype.

Fetal GITO often coexists with other ultrasound abnormalities. Polyhydramnios is considered a common development secondary to gastrointestinal obstruction, and it may be the result of impaired swallowing. In our cohort, polyhydramnios was noted in 41.4% of the cases, much lower than the frequency reported in most previous publications. This difference may be explained by the different timing of ultrasound scans. Similar to that reported by Orgul et al. (15) and Haeusler et al. (1), associated structural abnormalities were observed in 21.4% of the cohort. Among all the associated malformations, cardiac anomalies were believed to be the primary cause of mortality post-birth (38). In our study, eight fetuses had cardiac anomalies; of them, six revealed chromosomal abnormalities (case 4–6, 10, and 12–13, Table 3) and ended in TOP, one fetus with normal karyotype died 6 months after the surgery procedure, and for the rest, one with normal karyotype was terminated because of multiple cardiac malformations. Therefore, in addition to genetic evaluation, a detailed ultrasound scan is of great impact on pregnancy outcome.

In terms of pregnancy outcomes, consistent with most previous research studies, isolated GITO had a lower rate of TOP and a higher rate of normal development and survival than non-isolated GITO. The survival rates of fetuses with GITO differed in different studies (9, 15). We observed that 51.5% of the fetuses had normal development after surgery operations, similar to that found in the report by Gokcen et al. (15). It is generally acknowledged that prenatal detection of GITO usually depends on indirect ultrasound and/or MRI findings, but with inaccuracy in some cases. In our study, three cases were healthy at birth, suggesting misdiagnosis during the prenatal period. It is worth noting that the pregnancies with duodenal atresia/stenosis showed the highest rate of chromosomal abnormalities in this study, but that they also had the highest survival rate. It is mainly because the majority of them were cases of isolated duodenal atresia/stenosis, and this further highlighted the importance of associated ultrasound findings on the pregnancy outcome of GITO.

The limitation of our study is the small sample size and the fact that CMA was not performed on all the fetuses. Thus, the data may not be convincing enough. Future multiple center studies with a large sample size will hopefully lead to a better understanding of the genetic disorders and outcomes of GITO.

In conclusion, our experience confirmed the strong association between Down syndrome and fetal GITO, especially duodenal stenosis. CMA can effectively increase the detection rate of chromosomal aberrations. When fetal GITO is suspected, associated chromosomal abnormalities and fetal anatomy should be carefully evaluated for pregnancy management.

## Data Availability Statement

The original contributions presented in the study are publicly available. This data can be found here: accession number GSE203126, https://www.ncbi.nlm.nih.gov/geo/query/acc.cgi?acc=GSE203126.

## Ethics Statement

The studies involving human participants were reviewed and approved by Protection of Human Ethics Committee of Fujian Provincial Maternity and Children's Hospital. The patients/participants provided their written informed consent to participate in this study. Written informed consent was obtained from the individual(s) for the publication of any potentially identifiable images or data included in this article.

## Author Contributions

XW and LS prepared the original draft. QS, QG, and YL prepared the experiment. SX and NL performed data analysis. LX and HH revised the initial manuscript. All authors have reviewed and approved the final article.

## Funding

This study was supported by the Science Foundation of the Fujian Province, China (Grant No. 2021J01413) and Joint Funds for The Innovation of Science and Technology, Fujian Province (Grant Nos. 2020Y9135 and 2020Y9159).

## Conflict of Interest

The authors declare that the research was conducted in the absence of any commercial or financial relationships that could be construed as a potential conflict of interest.

## Publisher's Note

All claims expressed in this article are solely those of the authors and do not necessarily represent those of their affiliated organizations, or those of the publisher, the editors and the reviewers. Any product that may be evaluated in this article, or claim that may be made by its manufacturer, is not guaranteed or endorsed by the publisher.
