# Corrigendum: Chromosomal abnormalities and pregnancy outcomes for fetuses with gastrointestinal tract obstructions

**DOI:** 10.3389/fped.2022.976997

**Published:** 2022-10-26

**Authors:** Xiaoqing Wu, Linjuan Su, Qingmei Shen, Qun Guo, Ying Li, Shiyi Xu, Na Lin, Hailong Huang, Liangpu Xu

**Affiliations:** ^1^ Department of Medical Genetic Diagnosis and Therapy Center, Fujian Maternity and Child Health Hospital, College of Clinical Medicine for Obstetrics and Gynecology and Pediatrics, Fujian Medical University, Fuzhou, China; ^2^ Fujian Provincial Key Laboratory for Prenatal Diagnosis and Birth Defect, Fuzhou, China; ^3^ Department of Laboratory Medicine, Fujian Medical University, Fuzhou, China; ^4^ Department of Pediatrics, Guangxi Medical University, Nanning, China

**Keywords:** gastrointestinal tract obstructions, traditional karyotyping, chromosomal abnormalities, chromosomal microarray analysis, down syndrome, copy number variants

A corrigendum on Chromosomal abnormalities and pregnancy outcomes for fetuses with gastrointestinal tract obstructions By Wu X, Su L, Shen Q, Guo Q, Li Y, Xu S, Lin N, Huang H and Xu L. (2022) Front. Pediatr. 10: 918130. doi: 10.3389/fped.2022.918130

In the published article, there was a mistake in affiliation of author Na Lin. His affiliation has been corrected from “5 Medical Genetic Diagnosis and Therapy Center, Fujian Provincial Maternity and Child Health Hospital, Affiliated Hospital of Fujian Medical University, Fuzhou, China” to “1 Medical Genetic Diagnosis and Therapy Center Fujian Maternity and Child Health Hospital College of Clinical Medicine for Obstetrics / Gynecology and Pediatrics, Fujian Medical University, Fuzhou, Fujian, China.” and “2 Fujian Provincial Key Laboratory for Prenatal Diagnosis and Birth Defect, Fuzhou, Fujian, China.”

The authors apologize for this error and state that this does not change the scientific conclusions of the article in any way. The original article has been updated.

