# Supplementary material for: Chromosomal Abnormalities and Pregnancy Outcomes for Fetuses With Gastrointestinal Tract Obstructions
Source: Front Pediatr. 2022 Jun 6;10:918130. doi: 10.3389/fped.2022.918130 (PMC9245709; doi:10.3389/fped.2022.918130)
Supplement: Supplementary file 1 [file Table_1.pdf]

Supplementary Table 1

Case number	Maternal age	Gestation age at GITO initially diagnosed	Other ultrasound findings	Types of GITO	Karyotyping results	CMA results	Outcomes
E1851	26	27	-	Duodenal atresia/stenosis	N	N	Live birth, normal development after surgery
E2147	25	25	-	Jejunal or ileal atresia/stenosis	N	N	Live birth, normal development after surgery
E2275	28	27	-	Duodenal atresia/stenosis	N	N	Lost to follow-up
E3264	29	30	-	Jejunal or ileal atresia/stenosis	N	N	Live birth, normal development after surgery
P4963	25	25	-	Duodenal atresia/stenosis	N	N	Live birth, normal development after surgery
P4964	25	25	-	Duodenal atresia/stenosis	N	N	Live birth, normal development after surgery
E2240	34	28	Hydronephrosis	Duodenal atresia/stenosis	N	N	Live birth, normal development after surgery
P6332	40	22	-	Duodenal atresia/stenosis	47,XY,+21	arr[GRCh37] (21)x3	TOP
E2322	34	27	Small-for-gestational-age fetus	Jejunal or ileal atresia/stenosis	N	N	Live birth, died five months after surgery, with unknown cause
E2647	28	31	Polyhydramnios	Jejunal or ileal atresia/stenosis	N	N	Live birth, normal development after surgery
E3021	26	26	Polyhydramnios	Duodenal atresia/stenosis	N	N	Live birth, normal development after surgery

Supplementary Table 1

E3447	25	27	Ascites, Echogenic bowel	Jejunal or ileal atresia/stenosis	N	N	Live birth, normal development after surgery
E3675	20	27	Echogenic bowel	Jejunal or ileal atresia/stenosis	N	N	Lost to follow-up
P7041	28	23	-	Duodenal atresia/stenosis	N	N	Live birth, normal development after surgery
P8664	34	23	-	Esophageal atresia/stenosis	N	N	Live birth with no gastrointestinal obstruction syndrome and normal MRI
P8869	38	21	-	Jejunal or ileal atresia/stenosis	N	N	Live birth with no gastrointestinal obstruction syndrome and normal MRI
P2462	28	25	Polyhydramnios	Duodenal atresia/stenosis	N	N	TOP
R2995	31	22	-	Duodenal atresia/stenosis	N	arr[GRCh37]17q12 34440088-362433650×3 mat	TOP
P4291	29	22	Ventricular Septal Defects, persistent left superior vena cava，Single umbilical artery，Polyhydramnios	Duodenal atresia/stenosis	N	N	TOP
R3442	38	25	-	Esophageal atresia/stenosis	N	N	Live birth, normal development after surgery
P5128	35	22	Polyhydramnios	Esophageal atresia/stenosis	N	N	TOP

Supplementary Table 1

P7128	34	24	Polyhydramnios, Horseshoe kidney	Esophageal atresia/stenosis	N	N	Live birth, normal development after surgery
P7522	30	22	Ventricular Septal Defects, overriding aorta,pulmonary stenosis,Nasal bone dysplasia	Duodenal atresia/stenosis	47,XY,+21	arr[GRCh37](21)x3	Stillbirth
R801	29	23	Spina bifida	Anal atresia	N	N	Live birth, rehabilitation treatment after surgery
P8151	32	24	Polyhydramnios	Duodenal atresia/stenosis	N	N	Live birth, normal development after surgery
R1050	37	31	Polyhydramnios	Duodenal atresia/stenosis	N	N	Live birth, died six months after surgery, with unknown cause
R1267	30	27	Polyhydramnios, Single umbilical artery	Esophageal atresia/stenosis	N	N	Live birth, normal development after surgery
R3714	32	15	Polyhydramnios, Single umbilical artery	Esophageal atresia/stenosis	N	N	Live birth, normal development after surgery
R4089	26	23	Mild tricuspid regurgitation	Jejunal or ileal atresia/stenosis	N	N	Live birth, normal development after surgery
S24	33	25	Fetal growth restriction	Duodenal atresia/stenosis	N	N	Live birth, normal development after surgery

Supplementary Table 1

S60	30	29	Polyhydramnios, Single umbilical artery	Esophageal atresia/stenosis	N	N	Stillbirth
Z18	37	21	Ventricular Septal Defects, double superior vena cava, aberrant right subclavian artery, Ventriculomegaly,Single umbilical artery	Anal atresia	47,XY,+mar	arr[GRCh37] 22q11.1q11.21(16,888,899-18,649,190)x4	Died ten days after birth

TOP, termination of pregnancy; N, normal
